# The Risk of Drug-Drug Interactions with Paracetamol in a Population of Hospitalized Geriatric Patients

**DOI:** 10.1155/2020/1354209

**Published:** 2020-01-19

**Authors:** Lykke Ida Kaas Oldenburg, Kim Peder Dalhoff, Luana Østerdal Sandoval, Charlotte Vermehren

**Affiliations:** ^1^Department of Clinical Pharmacology, Copenhagen University Hospital Bispebjerg, Bispebjerg Bakke 23, DK-2400, Copenhagen, NV, Denmark; ^2^Department of Geriatric Medicine, Copenhagen University Hospital Bispebjerg, Bispebjerg Bakke 23, DK-2400, Copenhagen, NV, Denmark

## Abstract

**Aims:**

This study investigates the consumption of paracetamol and the risk of potential drug-drug interactions and assesses the clinical impact hereof in patients admitted to a department of geriatric medicine.

**Methods:**

A retrospective and longitudinal study was conducted in patients who had been receiving paracetamol upon or during hospitalization. The hospital files of the included patients were reviewed, including documentation of concomitant medications, diagnoses, biochemical values, and adverse incidents during admission. These parameters were used as a clinical follow-up when assessing a clinical probability impact of the identified drug-drug interactions.

**Results:**

In total, 104 patients were admitted during the study period. 91 (87.5%) of these (mean age 86 years) received a prescription or were treated with paracetamol. Of these, 10% were evaluated as being at risk of potential drug-drug interactions with paracetamol. Seven of the potential drug-drug interactions were related to treatments with warfarin, one with valsartan and one with phenytoin. Of the nine patients at risk, six did experience either abnormal biochemical values or potential related clinical incidents. Four patients experienced increased INR (range 3.2–4.6), of which one patient suffered from anaemia and one with hematemesis. Two patients experienced increased ALAT/ASAT (55/42 U/I and 87/51 U/I, both females). One experienced hypertension.

**Conclusion:**

A large majority of the patients in this study received treatment with paracetamol. Six patients were evaluated as having abnormal biochemical values or were experiencing clinical incidents during their hospitalization potentially related to the identified potential drug-drug interactions.

## 1. Introduction

The geriatric population is the fastest growing segment of our society, and this population is very heterogeneous [1, 2]. Pain is common among the elderly since they are more likely to suffer from arthritis, bone and joint disorders, and other chronic conditions [3]. Paracetamol is the most commonly used analgesic for acute and mild-to-moderate pain and is available without prescription [4–7] and is the first-line analgesic in Denmark [8] used as a maximum of four grams per day [9]. Elderly patients are often underrepresented or not included in clinical trials [10], and patients admitted to departments of geriatric medicine are assumed to be more frail and in greater risk of adverse events as they often are treated with concomitant medication due to associated diseases [11, 12]. These patients are often more vulnerable due to age-related changes in pharmacokinetics and pharmacodynamics [13]. This may lead to an increased risk of acquiring potential toxicity and drug-drug interactions (DDIs) [10, 12]. Furthermore, it has been demonstrated that age and sex are important factors affecting the pharmacokinetics of paracetamol. This results in higher concentrations of paracetamol with increased age and particularly elderly female patients [14]. Despite these findings, recommendation for reducing the maximum daily dosage (4 grams) in elderly patients has not been assessed to be necessary [2, 8, 15].

Several studies have reported and investigated pDDIs with paracetamol and the anticoagulant warfarin [16–21]. This interaction is assumed to be a pharmacokinetic interaction [19], resulting in a reduction of warfarin clearance [22] and hence increasing international normalized ratio (INR). This may lead to life-threatening bleeding. One such fatal DDI was reported in a Danish case report in 2015, when an 83-year-old man suffering from atrial fibrillation and back pain died from an untreatable intracerebral haemorrhage [23]. It has been suggested that decreased activity of CYP2E1 due to aging could increase the activity of paracetamol metabolised via CYP1A2 and CYP3A4 [24]. Thereby, competing with metabolism of *R-*warfarin which leads to inhibition of CYP2C9 that metabolizes *S*-warfarin. This can result in increased concentration of the *S*-isomer, which is five times as potent as the *R*-isomer [21, 24–26].

## 2. Aim of the Study

The aim was to investigate the use of paracetamol in elderly patients, who have been hospitalized in a department of geriatric medicine at a secondary hospital providing comprehensive health services. The focus was to identify the number of patients at risk of pDDIs with paracetamol and to provide a follow-up assessment of the clinical impact hereof by thoroughly investigating the patients' journals.

## 3. Method

### 3.1. Settings

The Department of Geriatric Medicine at Bispebjerg Hospital is a department with the capacity of 28 beds and a coverage area of appr. 400,000 citizens in an area that is part of greater Copenhagen and with a very diverse population. The department has a yearly admission of appr. 900 patients per year, whereas appr. 60% is admitted directly from the emergency department. Data for all admitted patients during appr. 5 weeks were collected and reviewed (in the period 1st of September to 10th of October 2016).

### 3.2. Screening of Patients

All electronic journals of patients aged 65 years or more, hospitalized in the Department of Geriatric Medicine at Bispebjerg and Frederiksberg, were screened for registration of any prescribed or consumed paracetamol upon or during their hospitalization. If paracetamol was used or prescribed upon or/and during admission, the patient's relevant biochemical values were registered; diagnoses upon and under admission and chronic and temporary diagnoses were identified by reviewing the anamneses and patient files. Also, clinical incidents during hospitalization were registered. These incidents were defined as the potential consequences of the DDIs described in the applied DDI databases. The remaining medications were also registered (medication history upon admission, prescriptions during hospitalization, active medication on the day of discharge, and administered medicine during hospitalization). The number of drugs was categorized and registered as the fifth level of ATC, both as needed medication (PRN) and regular prescription.

### 3.3. Data Management

The percentage of the patients receiving any treatment with paracetamol was calculated, including both the number of regular medications and PRN. For those who did receive any paracetamol treatment, the average administration of paracetamol per day per patient was calculated and compared toward the maximum recommended dosage.

The known pDDIs with paracetamol and *third-line* pDDIs were identified by using the databases *Micromedex* (MM), interaktionsdatabasen (ID), and *pro.medicin* (PM). Only pDDIs classified as *major* and *moderate* severity at MM and *critical* and *potential* problematic at ID were considered for this study.

A *third-line* pDDI was defined as a pDDI including the drug that also causes a pDDI with paracetamol. For instance, simvastatin-warfarin is a *third-line* pDDI because warfarin also can lead to a pDDI with paracetamol. This *third-line* pDDI may influence the pDDI between paracetamol and warfarin. A *third-line* drug was defined as the drug that had a pDDI with the same drug that was identified to have a pDDI with paracetamol. For instance, simvastatin is the *third-line* drug in the example of *third-line* pDDI. The patients at risk of a pDDI with paracetamol were identified, and subsequently, more thorough assessments of the patients were done. The assessments included an overall medication review of the patients' remaining medication.

Eventually, the patients at risk of pDDIs with paracetamol were evaluated due to any relation between the pDDIs with paracetamol and their biochemical values, diagnoses on admission or incidents noted in their journal during hospitalization. Incidents were identified according to key words in the applied DDI databases.

## 4. Results

During the data collection period, 104 patients were admitted to the Department of Geriatric Medicine and their patient files were reviewed. As seen in Table 1, 91 patients (87.5%) received paracetamol upon admission and/or during their hospitalization, of which 55% received it as a regular medication. The patients receiving paracetamol were as a median hospitalized for eight days. The median age was 86 years, and the majority were female (64.8%). The patients received several concomitant medications, with an increase of 28.5% of median prescribed medication from admission to discharge. Nine patients (10%) were identified to be at risk of a pDDI with paracetamol. No patients received more than recommended daily doses, but 26.4% received more than three grams per day as a mean. In addition, more than half of the patients, who received paracetamol as a regular medication at discharge, did receive one or more of other analgesics as regular medication—with oxycodone as the most frequently used add-on.

The different pDDIs with paracetamol are shown in Table 2, which also illustrates which pDDIs were listed in the three different databases ID, MM, and PM. Seven of the patients were identified at risk of the pDDI with paracetamol and warfarin. One patient was at risk of a pDDI with paracetamol and phenytoin. The last patient was at risk of a pDDI with paracetamol and valsartan.

As seen in Table 3, seven of the patients used paracetamol before admission according to their medication history obtained at admission. One patient did not obtain a medication history. The dose of paracetamol was not specified for another patient, and furthermore, it was not specified for four other patients if the prescription of paracetamol was as PRN or regular medication. Seven patients were discharged or transferred with an active prescription of paracetamol either as PRN or regular medication or in combination; one patient was paused, and another was discharged without paracetamol.

As seen in Table 4, the range of age of the nine patients at risk was 69 to 95 years, and eight of the patients were female. Six of the patients had a low haemoglobin value, three of them with a value between 5.0 and 6.0 mmol/l (moderate anaemia). Six of the patients had leucocytosis (indication of unspecific inflammation) with the highest value of 17.4 billion leukocytes per litre. Six of the patients had an estimated glomerular filtration rate (eGFR) below the reference value (indication of a poor function of the kidneys), two of which had an eGFR below 40 mmol/l. Only two patients had measures of alanine aminotransferase (ALAT) (indication of liver damage). Both patients had a value slightly above the reference with the highest value of 87 U/l. All patients had measures of aspartate aminotransferase (ASAT) (indication of damage to the liver and other tissues as muscles, pancreas, lungs), and the same two patients mentioned above did have a value slightly above the reference value. Five of the patients at risk of a pDDI with paracetamol and warfarin had sub- or supratherapeutic INR. Only one patient had a subtherapeutic INR of 1.7. The remaining patients had supratherapeutic values, with the highest value of 4.6. In addition, all the patients at risk of a pDDI with paracetamol had *third-line* pDDIs in the range of one to five other *third-line drugs.* The pDDI of paracetamol and warfarin can lead to bleeding and anaemia, one of the three patients who had moderate anaemia experienced bleeding and hematemesis, during the hospitalization. The pDDI of paracetamol and phenytoin can lead to insufficient effect of paracetamol and an increased risk of hepatotoxicity. The patient at risk of this pDDI had both an episode of pain breakout and as well a suspicion of hepatotoxicity mentioned in her journal. The pDDI of paracetamol and valsartan can lead to tachycardia and hypertension, and the patient at risk had hypertension mentioned in her journal. She was known to have irregular hypertension according to the hospital records.

In the aggregate, four of the nine patients experienced incidents during their hospitalization that could be related to the pDDIs with paracetamol.

As seen in Table 5, the patients at risk had in average six chronic diagnoses (range, four to nine) and 7 patients were transferred from the emergency department and two from an outpatient clinic.

Four of the patients that experienced increased INR (patients 2, 3, 6, and 7) were either admitted with or diagnosed during admission with an infection (three with urinary tract infection and one with pneumonia). The patient in risk of pDDI with phenytoin and paracetamol was hypotensive and had malnutrition.

To sum up, six patients had either influenced biochemical values (patients 2, 3, 6, and 7) and/or experienced incidents (patients 3, 5, 7, and 9) during hospitalization that relates to the outcomes described in the applied databases.

## 5. Discussion

This study shows that most patients hospitalized at a department of geriatric medicine received treatment with paracetamol, not surprisingly, indicating that many elderly patients experience pain. A previous systematic review described that, in Denmark in 2013, 23% of patients in age group 65–79 years and 45% in age group 80–89 years received prescribed paracetamol [27]. This indicates that patients in a department of geriatric medicine suffer from more pain than elderly in the remaining society. As several (26.4%) received more than three grams of paracetamol per day as a mean, it suggests that several elderly patients receive chronic pain treatment with paracetamol. The aforementioned review concluded that paracetamol as a chronic pain treatment showed minor efficacy and doubtful clinical relevance, suggesting that patients in chronic treatment should have their treatment reassessed [27].

Table 1 shows that the patients as an average received a lot of concomitant medication, supporting the assumption that elderly patients suffer from concomitant diseases and comorbidity. A previous study concluded that frail elderly patients had decreased liver function and decreased glucuronidation of paracetamol compared to fit elderly patients and hence decreased paracetamol elimination [28]. This indicates that frailty and not age is the most important factor, when evaluating paracetamol pharmacokinetics in elderly patients. The increase in prescribed medication at discharge compared to admission can be explained by either a lack of information in the medication history documented in the journals upon admission or new conditions to be treated with drugs.

The inconsistency between the applied electronic drug-drug interaction databases as seen in Table 2 is quite prominent and could be a great issue for the medication reviews performed by healthcare providers. This issue was observed in a Norwegian study as well, even though they used two other databases in their study [11].

To our knowledge, no previous studies with focus on pDDIs with paracetamol in the geriatric inpatients have been conducted. The present study showed that approximately 10% of elderly patients receiving paracetamol was at risk of pDDIs hereof. A Swedish study from 1993 described the risk of pDDIs with paracetamol in elderly outpatients, showing that 8.7% of outpatients were at risk of pDDIs with paracetamol [29]. The drugs found to interact with paracetamol in the Swedish study were carbamazepine, phenytoin, and phenobarbital. It is striking that none of the Swedish patients were treated with warfarin or that the pDDI with warfarin was not described. Especially, the DDI has been described several years before, for instance, in a clinical study in 1982 [30]. This is in contrast with the observations in the present study in which the interaction with warfarin was the most frequently and potentially dangerous interaction. This indicates that both patients and healthcare providers (physicians, pharmacists, etc.) should be trained in these pDDIs to strengthen patient safety. This can reduce the incidence of fatal and serious outcomes as previously observed in the Danish case report, described in the introduction [23]. The outcome of concomitant treatment with warfarin and paracetamol is very diverse in our study. An explanation can be that some patients did receive paracetamol as a regular medication ant their warfarin dosages has been adjusted to this, while some did receive PRN medication or initiated the paracetamol treatment when hospitalized. Previous studies have shown increased INR in stable warfarin patients who received two grams or more of paracetamol per day [17, 21]. Furthermore, the patient's *third-line drugs* and their dosage regime might be able to influence the outcome of the interaction between paracetamol and warfarin. Also, infections are thought to increase the risk of supratherapeutic INR with or without antibiotic exposure, but to our knowledge, this is only showed in a study that investigate patients receiving warfarin and who develop an acute upper respiratory tract infection [31]. The underlying mechanism is unknown, and the potential explanations mentioned in the article include reduced oral intake and decreased consumption of vitamin K-rich foods, the effect of paracetamol-containing cough and cold remedies that can increase the INR, or increased clotting factor catabolism associated with fever [31]. All in all, explanations not necessarily have anything to do with the general perception of an infection.

Despite no serious outcomes like severe bleeding or death, four of the investigated patients developed abnormal biochemical values or clinical incidents that could be caused by this well-documented DDI with warfarin and paracetamol. The heterogeneity of elderly patients can explain why some are more prone to this DDI than others. Patients who are *poor metabolizers* of the CYP2C9 will also have decreased clearance of warfarin, as the more potent *S-*isomer of warfarin is metabolised by this enzyme [26] and be more prone to this DDI. Due to this heterogeneity and various outcomes for patients in concomitant treatment with paracetamol and warfarin, a previous study recommended that more serious safety consideration should be given for patients at risk of this interaction [20]. The patient at risk of a pDDI with paracetamol and phenytoin experienced clinical incidents of both pain and decreased liver function. This patient was also identified as malnourished, and the decreased liver function was assessed to be associated with poor nutrition by the hospital physician. Phenytoin induces glucuronidation [32], as well as oxidation by inducing CYP3A4, which may lead to decreased area under the curve of paracetamol, due to an enhancement of first-pass metabolism of paracetamol [33]. This can lead to decreased analgesic effect. Theoretically, the induction of CYP3A4 increases the formation of NAPQI. This can increase the risk of hepatotoxicity due to a potential depletion of glutathione storage for further conjugation of NAPQI. This is to our knowledge only shown in patients who have taken an overdose of paracetamol, hence depleting glutathione storage [34–38]. Due to the patient's malnourishment and inflammation, she was likely to be at risk of depletion of glutathione storage [39]. In combination with her high daily dosage of paracetamol and regular dosage of phenytoin, it is likely that her incidents were related to the pDDI.

The last patient at risk of a pDDI was the patient receiving valsartan. During hospitalization, the patient experienced hypertension. A previous study showed a significant increase in blood pressure in patients treated concomitantly with paracetamol and valsartan, but the mechanism of the hypertensive effect is not fully understood [40]. The patient had a history of uncontrollable hypertension, which may be ascribable to the DDI or other unknown causative factors. The participants in the aforementioned study were administered one gram of paracetamol twice daily. They were excluded or withdrawn from the study if they were above 65 years of age and had uncontrolled hypertension. The patient in our study then seemed to be at a much higher risk of this pDDI because of her higher age, possible altered pharmacology, and uncontrollable hypertension. Particularly, this is because of the increased prescription dosage of paracetamol during hospitalization. This indicates that the healthcare providers had not identified or suspected the pDDI or assessed it as clinically relevant.

The knowledge of the widely used paracetamol's potential to interact with other drugs appears to be limited as none of the healthcare providers seemed to suspect the incidents to be related to the pDDIs with paracetamol, which might have been the case.

The finding and objective of our study was not to evaluate if the patients should have reduced or withdrawn their paracetamol treatment. Paracetamol is still the first-line analgesic even in patients at risk of pDDIs [18, 41]. In geriatric patients, untreated pain can have negative outcomes such as decreased quality of life and associated health issues like depression, anxiety, and sleep disturbances [3]. Thus, the intention of this study was to show that even though paracetamol is considered the safest choice, these pDDIs are important to keep in mind both for physicians, who prescribe it, and the pharmacy, who delivers it over-the-counter to the patients. The intention is also to make the prescriber think twice when prescribing the drug, a suggestion could be to monitor the effect after a suitable time of treatment, and potentially deprescribe the well-intended treatment in case of DDI findings, other adverse effects, or lack of effect.

A challenge is also when patients purchase paracetamol over-the-counter in ordinary shops. Due to free trade and a lack of required information, patients can encounter potentially dangerous situations with the drug.

The strength of this study is that several parameters have been investigated to assess patients at risk of a DDI with paracetamol. It was not only the patients' prescribed medications that were analysed, but also the administration of medication, the incidents during hospitalization, and relevant biochemical values were collected and analysed. These parameters were used as a clinical follow-up, providing a clinical probability impact of the identified DDIs.

The limitations were, as for all retrospective studies, the missing possibility to investigate relevant questions to the patients' medication and diagnoses and uncertainty or errors in information from the hospital's files. This study was made on a small number of patients, and a more extensive study is needed to support and empower our findings. This study was an exploratory study investigating the need and feasibility for a greater study to be conducted in the elderly patients consuming paracetamol.

## 6. Conclusion

A large majority of the patients admitted to a department of geriatric medicine at a large urban secondary hospital received treatment with paracetamol. Approximately, 10% of the patients treated with paracetamol were at risk of pDDIs with paracetamol, and warfarin was the most frequent drug to act with paracetamol, imposing a risk of a serious clinical incident. None of the patients experienced any serious outcomes, suggesting that heterogeneity and confounding factors as concomitant medication, *third-line* pDDIs, other diseases, inflammation, metabolism, and malnutrition can contribute to the outcome. Six patients were assessed to experience either influence of biochemical values or incidents during hospitalization that could be related to the identified pDDIs. This study highlights the importance of carefully reviewing the need of paracetamol in a vulnerable group of patients and always considering nonpharmacological treatment before beginning a chronic and long-term treatment; or in case of lack of effect in a long-term treatment, consider the possibility to deprescribe the drug to avoid serious adverse reactions due to interactions with other drugs and in general in order to avoid unnecessary polypharmacy and irrational medical treatment.

## Figures and Tables

**Table 1:** Data of the 91 patients receiving paracetamol.

Gender (female/male)	59/32
	Median (range)
Age (years)	86 (68–101)
Duration of hospitalization (days)	8 (1–31)
Number of other drugs upon admission^A,B^	7 (0–19)
Number of drugs at discharge^A^	9 (2–20)
Number of drugs, total exposure^A^	15 (4–29)

^A^Not including paracetamol. ^B^Eight patients did not have any registration of their medication history upon admission. The number of drugs upon admission was registered from the medication history described in the patients' journals upon the admission (not necessarily from the Department of Geriatric Medicine). The numbers of drugs at discharge and total exposure were registered from EPM and include both regular and PRN medication, and all prescriptions were registered except fluid infusions. The drugs were categorized in ATC codes. EPM = electronic patient module; PRN = as needed; pDDIs = potential drug-drug interactions; ATC = Anatomical Therapeutic Chemical Classification System.

**Table 2:** pDDIs described in the three different databases and the number of pDDIs for the 91 patients.

Potential DDI with paracetamol	ID	MM	PRO	Incidents number (%)
Warfarin	+	+	+	7 (7.7%)
Phenytoin	+	+	0	1 (1.1%)
Valsartan	+	0	0	1 (1.1%)
Isoniazid	+	++	0	0
Pneumococcal 13-valent vaccine	0	++	0	0
Imatinib	0	++	0	0
Pixantrone	0	++	0	0
Carbamazepine	0	+	0	0
Acenocoumarol	0	+	0	0
Lixisenatide	0	+	0	0
Zidovudine	0	+	0	0
Busulfan	0	+	0	0
Piperaquine	0	+	0	0
Diflunisal	0	+	0	0
Sulfinpyrazone	0	+	0	0
Aliskiren	+	0	0	0
Phenprocoumon	+	0	0	0

0 = pDDI is not mentioned. + = pDDI is marked as “potential problematic” and “moderate severity” for ID and MM, respectively. ++ = pDDI is marked as “critical” and “major severity” for ID and MM, respectively. PM does not classify DDIs. pDDI = potential drug-drug interaction; ID = interaktionsdatabasen.dk; MM = Micromedex; PM = pro.medicin.dk.

**Table 3:** The nine patients at risk of known pDDIs with paracetamol and their consumption of paracetamol.

Patient	Duration of hospitalization (days)	On admission (g)		Total administered		At discharge (g)		Maximum recommended dose complied
		Regular	PRN	g	g/day^D^	Regular	PRN
1	7	N/a	N/a	28	4	2	2	Yes
2	7	0	4	3	0.43	0	4	Yes
3	9	0	0	1	0.11	0	0	Yes
4	7	0	4	16	2.29	3	0	Yes
5	15	4^A^	N/a	50	3.33	0^C^	0	Yes
6	9	4^A^	N/a	36	4	4	0	Yes
7	31	4^A^	N/a	91	2.94	4	0	Yes
8	7	0	+^B^	14	2	3	1	Yes
9	8	1^A^	N/a	27	3.38	4	0	Yes

^A^It was not specified if the prescription was as PRN or regular medication. ^B^The amount was not specified. ^C^The prescription 4 × 1000 mg per day was paused the day of discharge. ^D^The mean administration in grams of paracetamol per day during hospitalization. pDDI = potential drug-drug interaction; PRN = as needed; g = gram; N/a = not available.

**Table 4:** For the nine patients at risk, biochemical values, pDDIs with paracetamol, *third-line* pDDIs, and incidents during hospitalization that relates to pDDIs with paracetamol.

Patient case		1	2	3	4	5	6	7	8	9
Gender (F/M)		M	F	F	F	F	F	F	F	F
Age (years)		71	91	86	82	69	95	89	95	87
Biochemistry	Reference value									
Hgb. (mmol/l)	M: 8.3–10.5 or F: 7.3–9.5	—	7.2	5.3	6.9	6.1	—	6.0	—	5.5
Leu. (x10^9^/l)	3.5–8.8	17.4	—	10.0	10.3	11.5	11.9	13.2	—	—
eGFR (ml/min)	>60	—	43	36	—	—	59	53	41	27
ALAT (U/l)	M: 10–70 or F: 10–45	N/a	N/a	N/a	N/a	55	87	N/a	N/a	N/a
ASAT (U/l)	M: 15–45 or F:15–35	—	—	—	—	42	51	—	—	—
INR	<1, 2, or 2-3^A^	—	3.5	3.8	1.7	—	3.2	4.6	—	—
Potential drug-drug interactions										
pDDI1	Paracetamol and warfarin	+	+	+	+		+	+	+	
pDDI2	Paracetamol and phenytoin					+				
pDDI3	Paracetamol and valsartan									+
Number of *third-line* pDDIs with										
	Warfarin	5	3	2	2		4	5	4	
	Phenytoin					5				
	Valsartan									1
Incidents at or during hospitalization^B^										
pDDI1	Bleeding or anaemia^C^			+				+		+
pDDI2	Pain					+				
	Hepatotoxicity					+				
pDDI3	Tachycardia									
	Hypertension									+

N/a = not available; — = if the value is within the reference value. The biochemical values mentioned are the most abnormal values during the hospitalization in the Department of Geriatric Medicine. Reference values are according to the mentioned value in OPUS. ^A^The treatment level during warfarin treatment with the indication of atrial fibrillation (given by sundhed.dk). ^B^The patient's diagnose upon admission or did the patient experience any incidents during hospitalization that could relate to the pDDI with paracetamol (according to the outcomes described in the applied databases). ^C^Anaemia is defined as haemoglobin 6.0 or less. pDDI = potential drug-drug interaction; *third-line* pDDIs: pDDIs with the same drug that is identified to have a pDDI with paracetamol; F = female; M = male; Hgb = haemoglobin; Leu = leucocytes; eGFR = estimated glomerular filtration rate; ALAT = alanine aminotransferase; ASAT = aspartate aminotransferase; INR = international normalized ratio; U = unit.

**Table 5:** Information of where the patients are transferred from their clinical diagnoses: chronic and temporary diagnoses as mentioned in their medication history and patients' files.

Patient	Transferred/admitted from	Admitted with	Chronic diagnoses	Temporary diagnoses (suspected during admission)	Incidents or altered biochemical values related to the pDDI
1	ED	Urinary tract infection Fever	Ischemic heart disease Chronic obstructive lung disease Lower urinary tract symptoms Atrial fibrillation Herniated disc		None

2	ED	Infection Fever	Atrial fibrillation Asthma Depression Cataract		Increased INR and decreased haemoglobin No incidents

3	ED	Fall Dizziness	Type 2 diabetes Atherosclerosis Renal impairment Hypertension Atrial fibrillation	Light anaemia Hypercalcemia Hypotensive No symptomatic urinary tract infection	Increased INR and decreased haemoglobin Incident: anaemia with no bleeding

4	ED	Confusion	Ischemic heart disease Cardiac insufficiency Paroxysmal atrial fibrillation Hypertension Hypercholesterolemia Angina pectoris	Insomnia Hyponatraemia	Increased INR and decreased haemoglobin

5	ED	Large loss of function Dehydration Hyponatraemia Malnutrition Abdominal pains	Hypertension Osteoporosis Back pain related to back collapse Postinfarction epilepsy	Hypotensive Pain outbreaks Obstipation	Increased ALAT and ASAT Incidents: pain and hepatotoxicity, light ascites

6	ED	Diffuse abdominal pain Urinary tract infection Cysts on liver Gallstones	Atrial fibrillation Hypertension Spinal stenosis Arthrosis Colostomy Recurrent urinary tract infection Parastomal hernia Tachy-brady syndrome	Hypertensive Dizziness	Increased ALAT and ASAT and INR No incidents

7	ED	Dehydration Emesis	Herniated disc Atrial fibrillation Chronic obstructive lung disease Osteoporosis Arthrosis Diverticulosis Benign kidney tumour Malnutrition Moderate mitral valve regurgitation	Pneumonia Fall during night Apnoea periods Urinary tract infection Delirium Hallucinations Hypotensive	Increased INR and decreased haemoglobin Incident: hematemesis

8	Outpatient clinic	Tachycardia	Atrial fibrillation Type 2 diabetes Depression Osteoporosis Gout	Atrial flutter Delirium Aggressive	None

9	Outpatient clinic	General impairment	Chronic leg ulcers Chronic obstructive lung disease Exertional dyspnoea Hypertension Chronic nephropathy Iron deficiency anaemia Hiatal hernia Peripheral oedema	Tired Nausea Stasis dermatitis Nephrogenic anaemia Dizziness	Decreased haemoglobin Incident: hypertension

ED = Emergency Department; pDDI = potential drug-drug interaction.

## Data Availability

The data used to support the findings of this study are restricted by the Danish Patient Safety Authority in order to protect patient privacy. Data are available from the corresponding author for researchers who meet the criteria for access to confidential data.
